# Correlates of Post-Stroke Brain Plasticity, Relationship to Pathophysiological Settings and Implications for Human Proof-of-Concept Studies

**DOI:** 10.3389/fncel.2016.00196

**Published:** 2016-08-05

**Authors:** Eduardo H. Sanchez-Mendoza, Dirk Matthias Hermann

**Affiliations:** Department of Neurology, University Hospital EssenEssen, Germany

**Keywords:** clinical translation, neuronal differentiation, neuronal plasticity, neuronal regeneration, restorative therapy

## Abstract

The promotion of neurological recovery by enhancing neuroplasticity has recently obtained strong attention in the stroke field. Experimental studies support the hypothesis that stroke recovery can be improved by therapeutic interventions that augment neuronal sprouting. However plasticity responses of neurons are highly complex, involving the growth and differentiation of axons, dendrites, dendritic spines and synapses, which depend on the pathophysiological setting and are tightly controlled by extracellular and intracellular signals. Thorough mechanistic insights are needed into how neuronal plasticity is influenced by plasticity-promoting therapies in order not to risk the success of future clinical proof-of-concept studies.

## Introduction

Following ischemic stroke, neuronal networks in the vicinity and at distance to the stroke are reorganized. Axons and axon collaterals are enabled to sprout (Reitmeir et al., 2011, 2012) and dendritic and synaptic processes are reshaped (Li et al., 2010; Overman et al., 2012) leading to an overall reorganization of the representational map that mediates the recovery of motor functions in a large number of settings ranging from rodents to primates and humans (Nudo and Milliken, 1996; Nudo et al., 1996; Wang et al., 2010; Overman et al., 2012). These reorganization processes enroll regions of the contralateral hemisphere (Mohajerani et al., 2011; Reitmeir et al., 2011) and have been shown to persist at least for several months post-stroke (Rossini et al., 2003; Sawaki et al., 2014). A wide variety of molecules are involved in these plasticity processes, including cell adhesion and guidance molecules, growth inhibitors, neurotransmitters and transport proteins (Bacigaluppi et al., 2009; Li et al., 2010; Sánchez-Mendoza et al., 2010; Reitmeir et al., 2011) that may activate or inactivate highly complex molecular pathways that enhance or inhibit neuronal sprouting and therefore local and remote connections (Li et al., 2010; Hermann and Chopp, 2012). Unfortunately, the natural capacity of the brain to rewire is insufficient. Though a degree of spontaneous sprouting exists (Li et al., 2010; Reitmeir et al., 2011, 2012) and some spontaneous recovery has been reported in humans (Duncan et al., 2000), neurological deficits persist in the large majority of stroke events even following localized or mild ischemic injuries (Hummel et al., 2005).

The intrinsic capacity of brain repair can be efficiently stimulated by exogenous therapeutic interventions, e.g., by physical exercise, delivery of growth factors, cell-based biologicals or pharmacological compounds, which in rodent and primate models of stroke were shown to enhance neurological recovery (Bacigaluppi et al., 2009; Reitmeir et al., 2011, 2012; Jaeger et al., 2015; Wang et al., 2016). Neurological recovery in the experimental setting can be defined as regain of lost function of the paretic limb as compared to a baseline defined previous to the stroke, which should not be confused with neurological compensation (Murphy and Corbett, 2009), in which other parts of the limbs (e.g., shoulder or the non-paretic limb) are recruited to complete a task. Neurological recovery and compensation can be discriminated by specific tests that allow the study of the paretic limb in isolation, e.g., pellet withdrawal in rodents or digit testing in primates that measure fine motor skills (Nudo and Milliken, 1996; Biernaskie et al., 2004), and tests measuring overall motor function, such as the rotarod, tight rope or hand grip tests (Doeppner et al., 2014a). There seems to be a critical time window after stroke in which various interventions, such as voluntary motor stimulation, pharmacological treatment or transcranial brain stimulation, can improve neurological recovery (Nudo et al., 1996; Biernaskie et al., 2004; Hummel et al., 2005; Sawaki et al., 2014; Wahl et al., 2014). In contrast to acute neuroprotective therapies, plasticity-promoting therapies have proven efficacy over weeks or even months post-stroke in animal and human studies.

Within this perspective article, we would like to briefly integrate some findings regarding brain remodeling and plasticity after stroke, elucidating: (a) structural surrogates of successful neurological recovery depending on the localization of ischemic lesions; (b) reorganization and plasticity processes of the cellular, subcellular and network level; (c) critical time windows for various therapeutic interventions; and (d) modes for the delivery of biologicals or drugs. We will shortly present (e) selected molecular signals that are likely mediators of plasticity processes, since we believe that understanding these signals is a major hallmark to prevent the failure of treatments in future clinical studies.

## Pathophysiological Setting Influences Neurological Recovery and Brain Plasticity

Ischemic stroke can affect both gray and white matter tissue, which invariably results in diverse patterns of brain injury and stroke recovery. In both cases, successful stroke recovery goes along with parenchymal tissue remodeling, involving: (a) the survival of neurons and axons in the surrounding of the ischemic lesion, which otherwise exhibit delayed degeneration; (b) the promotion of perilesional axonal, dendritic and synaptic plasticity; (c) the outgrowth of axons and axon collaterals at distance to the stroke; and (d) the modulation of astroglial and microglial responses, which may both promote or impede neuronal sprouting, depending on their activation state (Bacigaluppi et al., 2009; Li et al., 2010, 2014; Liu et al., 2010; Reitmeir et al., 2011, 2012). In a cornerstone study, Nudo and Milliken (1996) showed that local neuronal networks adjacent to the lesion can incorporate surviving neurons into their representational space. In this study, the authors described that neurons involved in distal limb movements before the stroke were recruited into networks involved in proximal limb movements after the stroke (Nudo and Milliken, 1996). Early motor training stimulation was found to retain and expand representational maps of the affected limb (Nudo et al., 1996).

**Figure 1:**
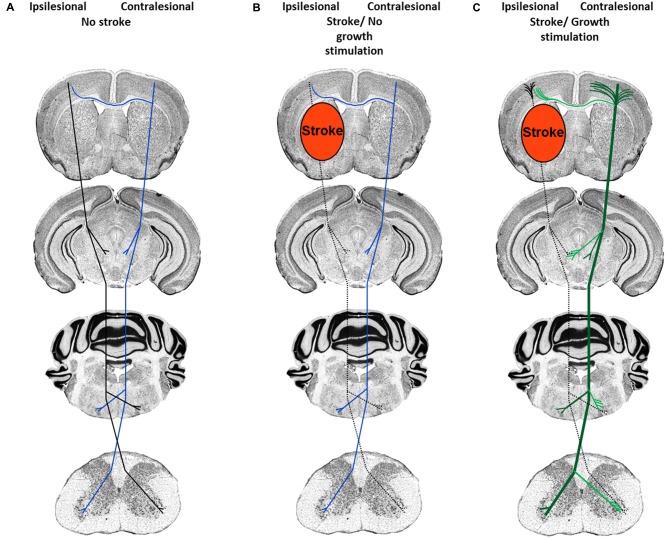
**Neuronal plasticity features of cortical neurons in response to stroke and neuronal growth stimulation. (A)** Organization of the corticospinal tract previous to the stroke. Ipsilesional fibers are depicted in black and contralesional fibers in blue. **(B)** After ischemic stroke induced by middle cerebral artery occlusion, pyramidal tract axons in the ipsilesional hemisphere degenerate (dashed lines), whereas contralesional pyramidal tract axons and dendrites exhibit scarce sprouting. **(C)** After growth stimulation, short-distance cortical dendrites exhibit abundant sprouting both ipsilateral (black) and contralateral (green) to the stroke, whereas long-distance axon collaterals grow out across the midline (exemplified here at the level of the red and facial nuclei and spinal chord) in direction to denervated target neurons. Post-stroke, the augmentation of contralesional axon collateral sprouting is accompanied by the improvement of motor and coordination deficits.

Depending on the severity of injury, damage to white matter may result in complete or incomplete fiber tract lesions. While in the case of complete fiber tract injury virtually no neuronal outgrowth is possible over larger distances, incomplete pyramidal tract injury, as induced by transient middle cerebral artery occlusion, does allow for the *de novo* formation of new terminal axon collaterals distal to the lesion site, as previously shown in studies in which plasticity of the ipsilateral and contralateral pyramidal tract was analyzed (Reitmeir et al., 2011, 2012). The delivery of the growth factors erythropoietin and vascular endothelial growth factor (VEGF) did not further augment the outgrowth of ipsilesional pyramidal tract fibers but induced the sprouting of midline-crossing contralesional pyramidal axon collaterals that accompanied functional neurological recovery (Figure 1; Reitmeir et al., 2011, 2012), showing a priming of the contralateral hemisphere for neuroplasticity responses that could be pharmacologically enhanced.

## Promotion of Neurological Recovery and Brain Plasticity: What to Do, When and How

Aspects of timing should be carefully addressed on restorative therapies to take better advantage of the early brain response to stroke. Several studies indicate a time window of about 1 week after the stroke in animal models and of 1 to 3 months in humans, in which the brain is particularly sensitive to the initiation of stimulating therapies (Biernaskie et al., 2004; Murphy and Corbett, 2009; Leasure and Grider, 2010; Zeiler and Krakauer, 2013; Wahl and Schwab, 2014; Dromerick et al., 2015; Ng et al., 2015). Thus, rats exposed to an enriched environment between 7–14 days, but not at 30 days after stroke, showed a sustained recovery of fine motor skills of the paretic limb (Biernaskie et al., 2004). Likewise, continuous training of the paretic limb started 5 days after the stroke lead to improvement of motor function in primates (Nudo et al., 1996). Moreover, mild voluntary exercise started 7 days after stroke improved somatosensory function in aged rats, while treadmill walking enhanced upper paretic limb skill in humans even months post-stroke (Ploughman et al., 2008; Leasure and Grider, 2010). Interestingly, non-invasive transcranial cortical stimulation, which regulates cortical neurotransmission by a non pharmacological approach, improves motor capacities both on acute, subacute and chronic stroke patients (Kang et al., 2016).

The delivery of biologicals or pharmacological agents also induces a time-dependent response of neurological function. Following neural precursor cell (NPC) delivery after middle cerebral artery occlusion in mice, both acute, sub-acute and post-acute intravenous NPC delivery up to 1 month post-stroke enhanced neurological recovery (Bacigaluppi et al., 2009; Doeppner et al., 2014a,b). While the neurological improvement was independent of the time-point of NPC delivery, the underlying mechanisms strongly varied depending on the timing of NPC grafting. Early delivery of NPCs up to 3 days after stroke potently induced neuroprotection, stabilized blood-brain barrier integrity, decreased brain inflammation and attenuated post-ischemic peripheral immunodepression, whereas post-acute NPC delivery at 28 days post-stroke induced more rigorous neuronal differentiation of grafted NPCs associated with enhanced angiogenesis and axonal plasticity (Bacigaluppi et al., 2009; Doeppner et al., 2014b).

Potential pitfalls related to the timing of treatment have been revealed after delivery of neutralizing antibodies against the axonal growth inhibitor NogoA, which, when administered prior to stroke, augmented ischemic injury in a mouse model of transient middle cerebral artery occlusion as a consequence of the early promotion of energy-requiring neuronal growth that resulted in the activation of cell death pathways. Importantly, the post-ischemic delivery of neutralizing NogoA antibodies did not have any injury-promoting effect (Kilic et al., 2010). That appropriate timing of physical and pharmacological therapies is crucial for the therapeutic responses induced was demonstrated in a combination study in which the delivery of neutralizing NogoA antibodies was combined with intense motor training. In this study, asynchronous NogoA antibody delivery immediately after stroke, but 2 weeks prior to intense motor training produced a remarkable recovery in fine motor function which was accompanied with contralesional corticospinal tract sprouting, while NogoA antibody delivery in parallel to motor training had no additive effect (Wahl et al., 2014). Time-windows should thoroughly be evaluated in animal studies during the preparation of subsequent clinical trials (Dromerick et al., 2015).

## Molecular Signals Associated with Post-Stroke Brain Plasticity

Neuronal sprouting tightly correlates with functional neurological improvement (Hermann and Chopp, 2012). Key molecular signals that control axonal and dendritic sprouting and dendritic spine density are Rho GTPases, phosphatidyl-inositol-3-kinase/Akt and cyclic nucleotides (Tashiro and Yuste, 2004; Yoshimura et al., 2006; Neukirchen and Bradke, 2011; Averaimo and Nicol, 2014; Spillane and Gallo, 2014). Are axons, dendrites and synapses consistently resuming the same ontogenetic programs that are activated during development (Cramer and Chopp, 2000) or are there also peculiar growth responses that differ between the developing and the stroke brain? In fact, post-stroke plasticity strongly depends on the re-expression of genes that mediate brain connectivity during normal development, which are down-regulated during adulthood under physiological states (Li et al., 2010). Yet, opposite to the developing brain, the post-ischemic brain represents an inflammatory environment that ultimately alters the final outcome of neuronal regrowth (Reitmeir et al., 2011, 2012; Lindau et al., 2014; Barbay et al., 2015). The different pathophysiological setting completely alters patterns of neuronal growth: while during ontogeny long-distance axonal connections are formed within the brain, post-stroke plasticity depends to a strong degree on terminal axon collateral sprouting (Reitmeir et al., 2011, 2012).

Interventions that modulate post-stroke plasticity share a number of common molecular merging points, of which Rho-GTPases deserve particular attention, since they control cytoskeleton remodeling (Leemhuis et al., 2010; Sun et al., 2012; Takeuchi et al., 2015). Rho-GTPases are a large family of proteins of which Rho-A/B, Rac and Cdc42, which have intensively been studied, have been related to axonal and dendritic sprouting in response to growth factor and neurotransmitter exposure. RhoA has an inhibitory effect on neuronal growth, whereas Rac-1 and Cdc42 have growth-promoting activity (Tashiro and Yuste, 2004; Ponimaskin et al., 2007; Leemhuis et al., 2010; Sun et al., 2012). Notably, both erythropoietin and statins, which promote contralesional pyramidal tract plasticity post-stroke and enhance functional neurological recovery in rodents (Reitmeir et al., 2011; Kilic et al., 2014), modulate Rho-GTPase activity. Erythropoietin inhibits Rho-A, ROCK-1 and ROCK-2 after optical nerve crush, promoting axonal growth (Tan et al., 2012), while it promoted axonal and dendritic growth on hippocampal cells through activation of the phosphatidyl-inositol-3-kinase/Akt pathway (Ransome and Turnley, 2008) that promotes microtubule polymerization (Yoshimura et al., 2006). Pravastatin inhibited the activity of RhoA in hippocampal neurons, thus stimulating dendritic and axonal branching. Deactivation of Nogo-66 receptor inhibited Rho-A, thus promoting neuronal growth (McGee and Strittmatter, 2003), while NogoA neutralization inhibited Rho-A and activated Rac1 and Rho-B (Kilic et al., 2010), thus enhancing post-stroke corticospinal tract plasticity (Lindau et al., 2014).

The timing of molecular pathway modulation is essential for stroke recovery. Following neutralizing NogoA antibody delivery, massive overactivation of the small Rho GTPases Rac1 and RhoB was noticed when the antibody was administered prior to stroke, the former of which overactivated the stress kinases p38 and Jun kinase-1/2 (JNK-1/2) thus activating cell death pathways (Kilic et al., 2010). The finding of exacerbated brain injury may not be an exception of the antagonization of a single molecule, i.e., NogoA, which is suggested by the observation that the deletion of the axonal guidance molecule ephrin-B3 enhanced post-ischemic neurogenesis, but at the same time increased infarct size resulting in poor neurological recovery (Doeppner et al., 2011). Accordingly forced paretic limb training caused secondary excitotoxicity that was reduced after administration of the NMDA receptor inhibitor MK801 (Humm et al., 1999). Since glutamate can modulate Rho-GTPase activity (Ponimaskin et al., 2007), uncontrolled glutamatergic transmission mediated by enhanced expression of VGLUT1 and NMDA receptor overactivation could lead to improper sprouting and delayed excitotoxicity (Sánchez-Mendoza et al., 2010). Importantly post-acute delivery of the uncompetitive NMDA receptor inhibitor memantine promoted neurological recovery and contralesional pyramidal tract sprouting (Wang et al., 2016), indicating that rebalancing of the excessive NMDA receptor stimulation at the right time-point indeed enhances post-stroke brain plasticity.

## Translation of Treatments to Human Stroke Patients

Some biologicals and pharmacological drugs have already passed preclinical proof-of-concept studies in animals and are presently undergoing first clinical studies in human stroke patients.

The neuropeptide cocktail cerebrolysin, which was shown to reduce apoptotic neuronal death *in vitro* (Gutmann et al., 2002) and to have a number of recovery-promoting, neuroprotective and restorative effects after focal cerebral ischemia in rodents, that included the promotion of axonal and dendritic remodeling (Hartbauer et al., 2001; Alcántara-González et al., 2012; Zhang et al., 2013), was recently reported to show beneficial effect on motor recovery when administered together with rehabilitation therapy in a small explorative randomized placebo-controlled study in human stroke patients (Muresanu et al., 2016). The Action Research Arm Test, not a global disability or dependence score, was the primary endpoint of this study. A larger multicenter trial needs to be performed to more definitely evaluate the efficacy of cerebrolysin.

The antidepressant fluoxetine, which is frequently used for treating post-stroke depressive syndromes, has been found to promote neurogenesis *in vitro* (Borkowska et al., 2015) and to extend the therapeutic window for brain rewiring after focal cerebral ischemia in mice (Ng et al., 2015). In a small randomized placebo-controlled study in human stroke patients, fluoxetine (20 mg per day) promoted motor recovery evaluated by the Fugl-Meyer motor scale over up to 3 months (Chollet et al., 2011). Global disability or dependence, was again not the primary endpoint of this study. A larger sufficiently powered efficacy study is urgently needed. In mice, fluoxetine was efficient in mice only when initiated 24 h, but not 7 days post-stroke, highlighting the relevance of therapeutic windows (Ng et al., 2015).

GSK249320 is an antibody that interferes the interaction of myelin-associated glycoprotein (MAG) and NogoA receptor. GSK249320 has been found to enhance neurite formation *in vitro* and reduce ischemic infarct size in mice, resulting in enhanced neurological recovery (Irving et al., 2005). Importantly, GSK249320 improved motor function after focal cerebral ischemia in squirrel monkeys (Barbay et al., 2015) and did not reveal any unfavorable effects in a safety study in healthy volunteers (Abila et al., 2013). Larger efficacy studies should be carried out in the future.

The phosphodiesterase inhibitor sildenafil, which increases intracellular concentrations of cGMP, a cyclic nucleotide involved in neuronal plasticity (Averaimo and Nicol, 2014), was shown to enhance the neurological recovery and neuronal plasticity in rat and mouse models of focal cerebral ischemia, stimulating axonal sprouting, neurogenesis, angiogenesis and oligodendrogenesis (Ding et al., 2008). Sildenafil was safe, when administered at a dose of 25 mg per day over 14 days in human subjects (Silver et al., 2009; Chen et al., 2011). A randomized multicenter study is warranted.

The delivery of the inverse agonist for the GABA_A_ α5 receptor L655708 was found to enhance neurological recovery after focal cerebral ischemia in mice via mechanisms including the reversal of excessive tonic inhibition of peri-infarct cortex (Clarkson et al., 2010). Following efficacy studies after proximal transient focal cerebral ischemia in mice and peripheral permanent focal cerebral ischemia in rats confirming enhanced neurological recovery using another GABA_A_ α5 receptor antagonist, S44819, proof-of-concept studies have been done in healthy humans, which confirmed the safety of S44819 over a wide dose range. A randomized multicenter study will be initiated in autumn 2016.

## Summary and Future Challenges

In view of the robust evidence that neurological recovery may be stimulated by therapeutic interventions that enhance neuronal plasticity, there is considerable hope that we may soon become able to use those therapies for enhancing neurological recovery in stroke patients. Stringent proof-of-concept studies are needed that comprise clear-defined “Go” and “No Go” decisions. For a proper idea of drug actions, morphological and molecular insights are required in the preparation of clinical trials that identify substrates of neuronal plasticity, which are subsequently targeted in stroke patients. In case of plasticity-promoting therapies, only well-defined proof-of-concept studies that define: (1) which type of tissue shall be targeted (previously ischemic, peri-lesional or lesion-remote tissue?), and (2) which structural features of neurons are influenced (neuronal survival, axon/axon collateral growth, dendrite/synapse formation?; see also Figure 1) and which is the most appropriate therapeutic window, will allow us to bring plasticity-promoting therapies into clinics.

## Author Contributions

Both the authors (EHS-M and DMH) wrote the article. All authors listed, have made substantial, direct and intellectual contribution to the work, and approved it for publication.

## Funding

This work was supported by the German Research Council (HE3173/3-1).

## Conflict of Interest Statement

The authors declare that the research was conducted in the absence of any commercial or financial relationships that could be construed as a potential conflict of interest.
